# Thermal Control Systems in Projection Lithography Tools: A Comprehensive Review

**DOI:** 10.3390/mi16080880

**Published:** 2025-07-29

**Authors:** Di Cao, He Dong, Zhibo Zeng, Wei Zhang, Xiaoping Li, Hangcheng Yu

**Affiliations:** 1 State Key Laboratory of Digital Manufacturing Equipment and Technology, School of Mechanical Science and Engineering, Huazhong University of Science and Technology, Wuhan 430074, China; caodi_hust@126.com (D.C.);; 2 School of Mechanical and Electronic Information, China University of Geosciences, Wuhan 430074, China

**Keywords:** projection lithography tools, thermal deformation, thermal control systems, thermal control structures, thermal control algorithms

## Abstract

This review examines the design of thermal control systems for state-of-the-art deep ultraviolet (DUV) and extreme ultraviolet (EUV) projection lithography tools. The lithographic system under investigation integrates several critical subsystems along the optical transmission chain, including the light source, reticle stage, projection optics (featuring DUV refractive lenses and EUV multilayer mirrors), immersion liquid, wafer stage, and metrology systems. Under high-power irradiation conditions with concurrent thermal perturbations, the degradation of thermal stability and gradient uniformity within these subsystems significantly compromises exposure precision. Through a systematic analysis of the thermal challenges specific to each subsystem, this review synthesizes established thermal control systems across two technical dimensions: thermal control structures and thermal control algorithms. Prospects for future advancements in lithographic thermal control are also discussed.

## 1. Introduction

**Figure 1:**
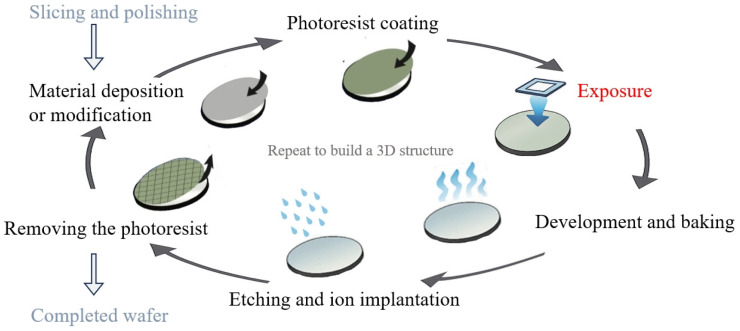
Photolithography in the recurrent process of making microchips.

Projection lithography tools are the core machines in semiconductor fabrication, responsible for transferring circuit patterns onto wafers with nanometer-scale precision. As illustrated in Figure 1, while the lithography process involves multiple steps such as photoresist coating, baking, and development, the exposure stage is the most critical for defining pattern resolution and placement accuracy [1]. Among these tools, advanced DUV and EUV systems represent the state of the art in high-resolution patterning.

**Figure 2:**
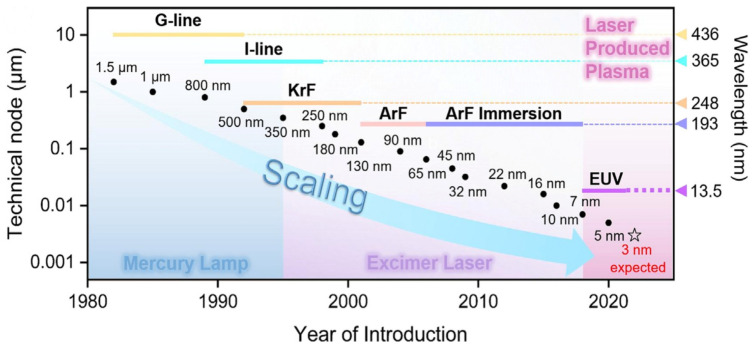
Scaling of technical nodes with the development of the semiconductor industry [2].

As shown in Figure 2, lithography technologies have evolved from i-line systems to advanced DUV and EUV platforms in response to the continuous demand for finer patterning and device scaling. This review focuses on the thermal control of modern projection lithography tools, particularly deep ultraviolet (DUV) and extreme ultraviolet (EUV) systems, which together form the technological backbone of current and next-generation high-resolution patterning.

Earlier-generation tools such as i-line systems are not discussed in detail, as their thermal control requirements—such as reticle- and wafer-stage temperature regulation or lens cooling—are largely inherited by early DUV platforms. As such, DUV systems serve as a more representative base for tracing the progression of thermal control strategies, especially given their direct technological evolution into EUV systems.

**Figure 3:**
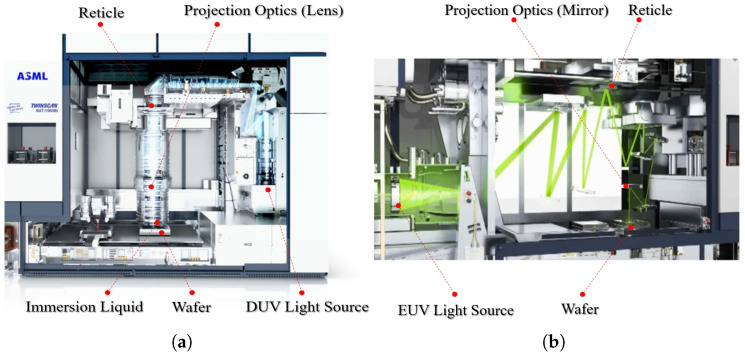
Schematic diagrams of subsystems for lithography tools. (**a**) TWINSCAN NXT:1960Bi DUV lithography tool (ASML, Veldhoven, The Netherlands); (**b**) TWINSCAN NXE:3100 EUV lithography tool (ASML, Veldhoven, The Netherlands).

A projection lithography tool comprises multiple high-precision subsystems, including the light source, reticle stage, projection optics (using DUV refractive lenses or EUV multilayer mirrors), immersion liquid module, and wafer stage, as illustrated in Figure 3. These precision subsystems operate under tight performance constraints and are sensitive to thermal disturbances. Temperature fluctuations along the optical path can degrade imaging performance through effects such as optical aberrations, stage deformation, or refractive index drift. Even nanometer-scale thermally induced displacements may cause overlay and focus errors [3,4]. As lithography has evolved from DUV to EUV, thermal management has become increasingly critical to system design and performance stability.

In DUV lithography systems, thermal disturbances arise from multiple sources across the optical and process chains. Excimer laser operations generate heat that raises the temperature of inert gas media, affecting the spectral stability of the light source [5]. Projection lenses absorb ultraviolet radiation, leading to thermomechanical deformation and wavefront aberrations [6]. In immersion lithography, temperature fluctuations in the ultrapure water alter its refractive index, degrading image fidelity [7,8]. Both the reticle and the wafer, directly exposed to high-power illumination, suffer from thermal deformation—reticles through absorption-induced expansion, and wafers through a combination of exposure heating, liquid evaporation, and thermal diffusion [4,9]. Interferometric systems used for stage positioning are also sensitive to air temperature and refractive index drift, introducing nanometer-scale misalignment [3]. These combined effects highlight the critical role of thermal management in preserving overlay accuracy and imaging stability across DUV subsystems.

EUV lithography introduces new thermal challenges due to its distinctive system architecture, including plasma-based light sources, fully reflective projection optics, and operation in a high-vacuum environment. Unlike DUV systems that use excimer lasers and transmissive lenses, EUV tools employ discharge-produced plasma (DPP) or laser-produced plasma (LPP) sources that emit 13.5 nm radiation. These sources exhibit extremely low conversion efficiency, resulting in substantial heat accumulation in collector mirrors and tin-droplet generators, which compromises plasma stability and component lifetime [10,11,12]. To mitigate absorption losses at EUV wavelengths, projection optics rely exclusively on multilayer Bragg mirrors rather than refractive lenses. However, each mirror still absorbs part of the incident energy, and in the absence of convective cooling under vacuum conditions, this leads to localized temperature increases and thermoelastic deformation of mirror surfaces [13]. Meanwhile, both the reflective reticle and the wafer are exposed to high-power EUV illumination: the reticle, composed of sensitive multilayer coatings, must maintain extreme flatness to avoid pattern distortion, while the wafer accumulates heat over repeated exposures and suffers from reduced thermal dissipation due to vacuum constraints [14,15]. Furthermore, although the vacuum environment removes refractive index instability—beneficial for interferometric and encoder-based stage metrology—it simultaneously exacerbates thermal buildup and complicates dynamic temperature control. These factors collectively impose significantly higher demands on thermal control in EUV systems compared to DUV, especially as critical dimensions shrink below the 5 nm node. Given these challenges, effective thermal management has become essential to sustaining pattern fidelity, overlay accuracy, and component reliability in next-generation lithography tools.

In addition to thermal regulation, mechanical compensation methods are also widely employed to mitigate thermally induced exposure errors. These include both shape adjustment techniques, such as deformable mirrors actuated by piezoelectric, electrostatic, or Lorentz forces [8,13,16,17,18,19]; and rigid-body pose regulation strategies, such as dynamic adjustments of wafer- and reticle-stage positions or lens actuator setpoints to offset thermal deformation [20,21,22,23]. These mechanical methods play a complementary role in exposure stability but are not the primary focus of this review.

Beyond mainstream semiconductor manufacturing, DUV and EUV optical lithography techniques have also enabled a range of high-precision applications in emerging photonic and nanostructure fabrication fields. Notable examples include the use of optical lithography for master mold fabrication in nanoimprint lithography (NIL) [24,25,26], all-glass metalenses for visible-wavelength photonics [27], and dielectric photonic crystals for super-resolution photolithography [28,29]. These applications impose similarly stringent requirements on pattern fidelity and thermal stability, particularly during the creation of high-resolution functional templates. Acknowledging these fields further emphasizes the critical role of advanced thermal control strategies, potentially extending their impact to a broader range of optical and photonic systems.

This review presents a structured survey of thermal control strategies in DUV and EUV lithography tools. Recent developments are broadly categorized into structural implementations and algorithmic approaches, examined across six critical subsystems: the light source, reticle, projection optics, immersion liquid, wafer stage, and metrology. For each subsystem, thermally induced errors and associated challenges impacting imaging performance are analyzed, followed by a review of existing thermal control structures and algorithms. Future research directions are discussed in the final section.

## 2. Thermal Control of Light Source Systems

### 2.1. Thermally Induced Errors and Challenges

**Figure 4:**
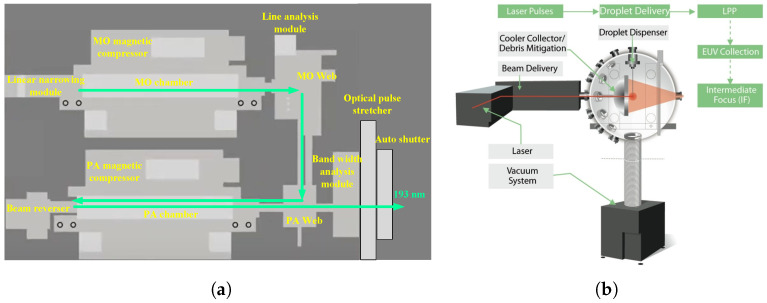
Representative source architectures for DUV and EUV lithography: (**a**) MOPA dual-cavity excimer laser system [32]; (**b**) LPP source system [10].

Thermal errors in lithography light sources primarily arise from substantial energy dissipation during high-energy photon generation. Figure 4 illustrates the structural schematics of a DUV excimer laser and a LPP EUV source, representing typical light source configurations in DUV and EUV lithography systems, respectively.

In DUV excimer laser systems (Figure 4a), the Master Oscillator (MO) and Power Amplifier (PA) convert a significant portion of input electrical energy into heat during discharge excitation [30]. This results in density fluctuations of the gaseous medium and thermal expansion of the optical elements. These thermal effects manifest as spectral shifts, wavelength instability, and pulse energy variations, which degrade critical dimension (CD) uniformity and overlay accuracy [5,31].

In LPP EUV sources (Figure 4b), a CO_2_ laser irradiates tin droplets to generate high-temperature plasma, with radiation intensity reaching several hundred watts. The collector mirror, responsible for EUV radiation collection and focusing, must maintain sub-nanometer figure accuracy under intense radiative heat load. Thermal deformation of the mirror leads to wavefront aberrations and degraded image resolution [10]. Simultaneously, the tin-droplet generator is exposed to high heat flux, and unstable temperature control can induce thermal fatigue or evaporation instability, causing fluctuations in droplet size and frequency, which compromise plasma excitation efficiency and EUV output power [12].

In DPP EUV sources, a hollow collector structure comprising multiple mirrors is typically employed to enhance light collection efficiency. However, thermal cycling induces coating degradation, accumulated stress, and decreased reflectivity [11]. In addition, gas temperature instabilities within the discharge chamber affect plasma uniformity and radiation consistency.

Multiple critical components within light source systems require high-precision thermal control: MO and PA chambers in DUV systems; collector mirrors, tin-droplet generators, and vacuum chambers in EUV systems. The thermal stability of these components directly determines plasma excitation efficiency, wavelength consistency, and imaging quality. Stable operation requires minimizing temperature fluctuations, suppressing thermal gradients, and preventing local overheating across varying loads and environmental conditions.

Key thermal control challenges include achieving high-spatial-temperature uniformity and fast thermal stabilization under high-repetition plasma excitation; designing vacuum-compatible cooling systems without compromising sealing or cleanliness; and coordinating distributed thermal sources such as collector mirrors, tin sources, and chambers through local regulation and global thermal field optimization.

### 2.2. Thermal Control Structures

**Figure 5:**
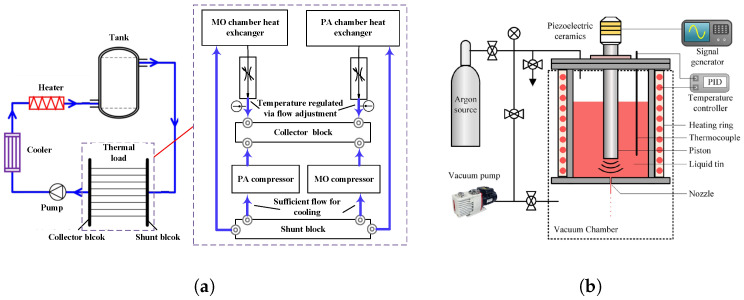
Representative thermal control structures: (**a**) Excimer laser thermal control system [33]; (**b**) LPP tin-droplet generator thermal control system [12].

Various thermal management structures have been developed to address these challenges. In DUV excimer lasers, water cooling remains the primary method. Heat exchangers, temperature sensors, and servo-controlled valves regulate coolant flow through emission zones, enabling targeted cooling of the MO and PA chambers [33,34,35], as illustrated in Figure 5a.

In contrast, EUV sources—particularly discharge-produced plasma (DPP) sources—require embedded thermal pathways to manage intense localized heating and maintain optical stability. For example, metallic cooling channels are embedded within mirror housings to enable uniform heat extraction and suppress thermal deformation [11]. A representative collector uses a dual-shell structure: the inner shell acts as the reflective EUV surface, while the outer shell provides mechanical support and contains dedicated cooling circuits. Thermocouples installed between the shells monitor temperature gradients and balance flow among channels. A dual-loop cooling configuration is typically adopted to manage the thermal loads of both the collector and the surrounding vacuum chamber, maintaining collector temperatures within 20–25 °C (Figure 5b).

For LPP sources, peripheral modules (e.g., vacuum enclosures and diagnostic flanges) often utilize double-walled circulation channels for cooling [36]. However, direct water cooling is impractical for certain vacuum-integrated components (especially the collector) due to sealing constraints. To overcome this, gas-based cooling is adopted. Actively regulated gas flows, governed by feedback control, ensure thermal uniformity of sensitive mirrors while maintaining vacuum compatibility. Under a 250 W thermal load, temperature rise can be limited to below 0.8 °C, and the intermediate focus (IF) spot size remains stable at 0.1 mm [10,37].

Furthermore, resistive heaters paired with closed-loop feedback control are commonly used to maintain tin-droplet generator temperatures at approximately 250 °C [12]. These tailored structures enable localized heating or cooling according to functional requirements, as shown in Figure 5b.

### 2.3. Thermal Control Algorithms

Thermal control algorithms are critical to fully exploit the aforementioned hardware capabilities. Proportional–Integral–Derivative (PID) control remains widely adopted in commercial systems. Initial designs employed standard PID controllers for regulating excimer laser chamber temperature [34], followed by improvements using Smith–PID predictive control to reduce overshoot and settling time [35]. More recent developments have introduced dual-input nonlinear PI controllers to enhance discharge chamber stability [33].

In more complex EUV systems, finite element modeling (FEM) and thermomechanical simulations are employed to predict mirror deformations and optimize coolant flow distribution. These models help balance thermal gradients during high-frequency operations and have demonstrated the ability to maintain component temperatures within 20–25 °C under coolant inlet temperatures of 18.6 °C [11].

While coordinated multi-objective thermal control strategies are not explicitly defined in the literature, key components such as collectors, vacuum chambers, and droplet sources are already operated with independent thermal loops. This distributed architecture provides a foundation for multi-loop control frameworks. Future strategies are trending toward “localized regulation + global field optimization” to precisely coordinate multiple thermal zones.

Moreover, in vacuum environments where water cooling is impractical, gas-based control strategies coupled with real-time sensing have been deployed to maintain thermal stability while ensuring vacuum compatibility [10].

Through integrated sensing, modeling, and closed-loop feedback, these algorithms enable robust thermal regulation of complex modules and effectively suppress opto-thermal errors under extreme heat loads.

### 2.4. Summary and Outlook

Thermal control of lithography light sources faces multiple challenges, including the superposition of high thermal fluxes from various sources, complex structural constraints, and heat transfer limitations in vacuum environments. In recent years, substantial progress has been made: structurally, collector mirrors and vacuum chambers often adopt dual-shell designs or embedded cooling channels, while feedback-regulated gas-cooling pathways have been developed for LPP systems. On the control side, algorithms have evolved from classical PID to predictive and nonlinear multi-input strategies, enabling both localized temperature zone regulation and preliminary coordination of the global thermal field.

Despite these advancements, several limitations remain. In excimer laser systems, thermal management still primarily relies on water-cooling structures and closed-loop PID control based on temperature feedback. While these approaches improve short-term stability, they respond sluggishly to dynamic thermal disturbances such as high-repetition-rate pulsed lasers or chamber aging effects. Although Smith-predictive compensation enhances stability in systems with delays, it lacks the capability to model thermal inertia dynamics and is ill-suited for managing spatially non-uniform heat sources.

For EUV light sources, the focus is placed on thermal control of vacuum-enclosed components such as collector mirrors and tin-droplet generators. Existing forced-liquid cooling methods are constrained by sealing challenges, mechanical vibrations, and component replacement difficulties. Therefore, further investigation is needed into alternative liquid-cooling loops and heat exchanger configurations that ensure stability and integrity during operation.

Gas cooling, while effective in laboratory-scale setups, presents uncertainties in large-scale production systems. Its compatibility with high-throughput EUV source environments—particularly in terms of spatial constraints, contamination control, and ease of integration—remains to be demonstrated.

## 3. Thermal Control of Reticle-Stage Systems

### 3.1. Thermally Induced Errors and Challenges

Thermal deformation of the reticle is a significant contributor to overlay errors in high-volume lithography. During exposure, the reticle—particularly those with low transmittance—absorbs optical energy and undergoes a non-uniform temperature rise, leading to thermomechanical expansion and image field distortion. In DUV systems, this results in persistent barrel-shaped overlay errors, which may not be fully corrected even after alignment compensation [21,22].

Compared to DUV, EUV lithography introduces substantially greater thermal challenges due to higher source power, increased pattern complexity, and limited heat dissipation in vacuum environments. The reticle becomes one of the most thermally stressed components in the system, making its thermal control a critical focus of recent research on distortion compensation and overlay accuracy improvement [38].

Under 13.5 nm EUV irradiation, energy is absorbed and accumulates over time, generating complex, nonlinear thermal gradients across the reticle. Without active thermal control, this cumulative heating leads to progressive overlay degradation across multiple wafers [38,39].

The core challenge lies in maintaining both low absolute temperature and high thermal uniformity under dynamically varying spatial and temporal heat loads. Conventional open-loop cooling or reticle-stage alignment-based compensation strategies are inadequate to address these complexities. Instead, model-driven thermal control approaches are increasingly required to ensure reticle stability.

### 3.2. Thermal Control Structures

To suppress overlay errors induced by thermal deformation, targeted thermal management strategies have been implemented on both the reticle itself and its mounting chuck, with specific adaptations for DUV and EUV systems. In DUV lithography tools (e.g., the NXT:1980Di platform), active airflow is directed across the reticle surface during scanning. Experimental results show that this technique can reduce the thermal deformation amplitude to approximately 40% of that observed without airflow control [22].

In EUV scanners, the reticle is electrostatically clamped onto a Zerodur-based chuck. To enhance thermal conduction while avoiding mechanical stress, sparse support points between the reticle and chuck are filled with hydrogen gas, thereby increasing thermal contact efficiency [20]. The chuck integrates internal liquid-cooling channels to efficiently extract heat.

Considering that EUV systems operate in vacuum environments where conventional convective cooling is infeasible, radiative heat transfer becomes the dominant dissipation mechanism. According to [40], a thin film of inert gas (e.g., nitrogen or argon) is retained near the reticle surface. This gas layer does not facilitate convection but instead enables thermal creep cleaning and limited thermal coupling at the reticle–chuck interface.

**Figure 6:**
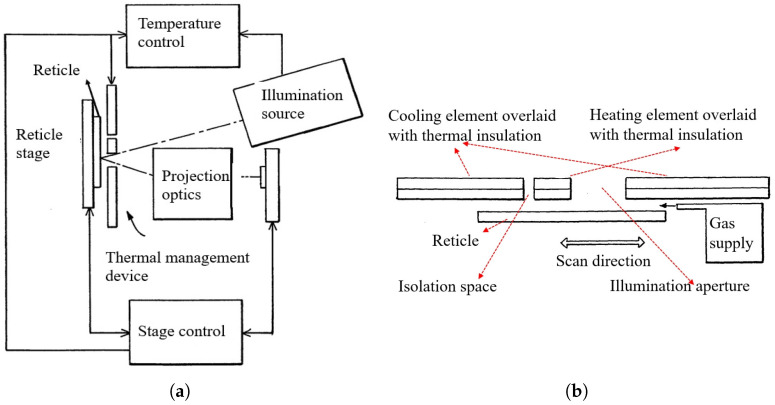
EUV lithography tool with integrated thermal management device [40]: (**a**) overview of the system; (**b**) detailed structure of the thermal compensation module.

Furthermore, to dynamically compensate for local thermal distortion caused by patterned illumination, the same patent proposes integrating spatially and temporally tunable thermal actuators—such as micro-heaters and cooling elements—around the reticle. These actuators provide localized, precise thermal compensation during scanning, thereby preserving image fidelity under non-uniform exposure conditions. A schematic of this concept is shown in Figure 6.

### 3.3. Thermal Control Algorithms

**Figure 7:**
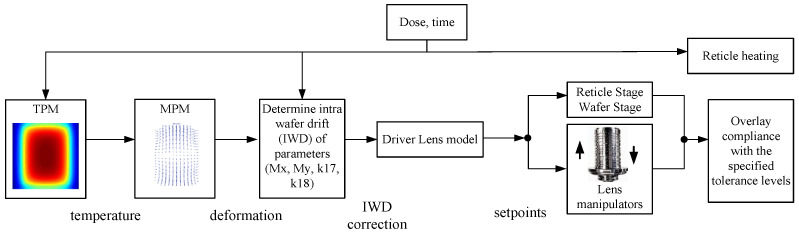
RHEC control concept. The thermo-dynamic prediction model (TPM) estimates the reticle temperature field during wafer exposure and enables feedforward correction based on the predicted overlay errors [22].

To achieve predictive control of the reticle temperature field under varying exposure conditions, researchers have proposed a range of model-based thermal error compensation strategies for real-time suppression of thermally induced deformation. One such approach is the Reticle Heating Error Correction (RHEC) control framework, as redrawn in Figure 7, which integrates a thermo-dynamic prediction model (TPM) into the system’s control loop. The TPM establishes a mapping from thermal input to overlay error and enables feedforward compensation by adjusting the posture of the reticle stage and projection optics [22].

As EUV system power continues to rise and overlay tolerances tighten, static models have become insufficient. To address this, advanced control algorithms incorporate Kalman filtering to fuse real-time thermal sensor data with a multi-physics model (MPM). This enables dynamic prediction and compensation of time-varying thermal distortions [41].

Although a fully closed-loop thermal control system for the reticle stage has not yet been realized, the thermal prediction models developed in systems such as RHEC already form a solid foundation for next-generation controllers. These models not only quantify the deformation response to spatially non-uniform thermal inputs but can also be integrated with feedforward regulation and model predictive control (MPC) frameworks to form predictive thermal control systems capable of meeting future mK-level stability requirements.

### 3.4. Summary and Outlook

In summary, the thermal control of the reticle system is implemented through active airflow in DUV tools, hydrogen-enhanced conduction and embedded cooling in EUV scanners, and model-based algorithms for predictive compensation of thermal distortion. However, due to the reflective structure of EUV reticles, direct temperature sensing near the patterned surface remains difficult. Most systems rely on sensors located at the backside or chuck edge, limiting the response to localized thermal variations.

Additionally, slow thermal conduction restricts feedback bandwidth, making real-time disturbance rejection in patterned regions challenging. Many thermal prediction models remain offline tools, not yet integrated into closed-loop control.

Future directions should focus on co-optimizing modeling, sensing, and actuation with thermal control performance as the target. Predictive control commands based on models like RHEC could be applied to localized thermal actuators, facilitating fully closed-loop, real-time thermal compensation systems.

Notably, the reticle and wafer stages share similar sources of thermally induced errors, as well as analogous design and control challenges. Detailed strategies for the wafer stage can be cross-referenced in Section 6 for further insight.

## 4. Thermal Control of Projection Optics Systems

### 4.1. Thermally Induced Errors and Challenges

Thermally induced distortions in projection optics are among the primary factors limiting lithographic imaging performance. These distortions result primarily from exposure-induced heating and off-axis illumination, which lead to temperature-dependent refractive index variations and thermomechanical deformation of optical elements.

In DUV systems, 193 nm irradiation causes symmetric wavefront aberrations in fused silica lenses, typically resembling convex lens deformation [42,43]. In contrast, EUV systems suffer from asymmetric heating of mirrors due to patterned reticle illumination, resulting in complex, non-rotationally symmetric deformations. These distortions degrade imaging fidelity and compromise overlay precision [13,44].

Quantitative studies have revealed the extreme thermal sensitivity of projection optics. Liu et al. [45] reported that, under thermal steady state, the M5 mirror in an EUV system can experience a temperature rise of up to 8.48 °C and temporal oscillations of 1.44 °C, corresponding to nanometer-level surface figure errors. Laufer [46] estimated that in order to constrain image drift to below 1 nm during a 60-s exposure, the temperature variation along the 2-m-long projection optical bench (POB) must not exceed $4.2\times 10^{-5}$ °C/min. This estimation, based on a structural coefficient of thermal expansion (CTE) of 15 ppm/K and a unitary coupling ratio between thermal deformation and image shift, reflects the extreme thermal sensitivity of EUV systems, where multilayer mirrors absorb 35–40% of incident EUV energy and are subjected to substantial and highly non-uniform thermal loads.

In addition to temporal stability, spatial temperature uniformity across mirror surfaces is equally critical. Thermal gradients must be rigorously controlled to prevent surface figure errors. For instance, Veldman et al. [47] established a ±3.5 °C tolerance threshold for mirror temperature differentials to maintain surface figure stability.

Thermal control of projection optics must not only address temperature drift and local deformation but also overcome a range of engineering challenges. First, the high-vacuum environment of EUV systems renders convective heat transfer mechanisms ineffective, constraining the design freedom of cooling structures. Second, the spatial distribution of heat load across multilayer mirrors is highly non-uniform and dynamically modulated by the reticle pattern, necessitating actuation mechanisms capable of fast, spatially resolved responses. Furthermore, the control system must accommodate delay compensation and disturbance rejection in order to achieve mK-level temperature stability. Together, these factors define the core challenges in the thermal management of projection optics, spanning structural design, actuator layout, and advanced control algorithms.

### 4.2. Thermal Control Structures

Thermal control structures in projection optics are designed to suppress imaging degradation caused by heat-induced optical distortions. Depending on the system architecture and ambient environment, two complementary mechanisms are commonly employed: conduction-based cooling using water jackets for bulk heat removal and infrared (IR)-based thermal compensation for precise, localized actuation. DUV lithography systems primarily rely on water jacket cooling, although IR-based compensation has also been explored. In contrast, EUV systems, operating in vacuum and utilizing multilayer reflective mirrors, predominantly adopt IR-based strategies for fine thermal regulation. Representative implementations are summarized below.

#### 4.2.1. Water Jacket-Based Cooling Structures

In DUV lithography systems, projection optics typically consist of refractive lens assemblies, and their thermal regulation primarily relies on convective cooling structures to achieve sub-Kelvin temperature stability. Chen et al. [48] proposed a compact water jacket cooling structure. In this design, 2 mm thick aluminum cooling tubes are tightly arranged along the outer surface of the lens. A 0.8 mm air gap is introduced between the cooling tubes and the lens body to create an indirect heat exchange path, effectively preventing direct contact between the coolant and the optical components. To enable real-time monitoring and feedback regulation, 13 thermistors are embedded within the structure. Additionally, surface-mounted heaters are integrated on the lens to provide fine thermal adjustments, thereby improving both the response speed and uniformity of the cooling structure.

**Figure 8:**
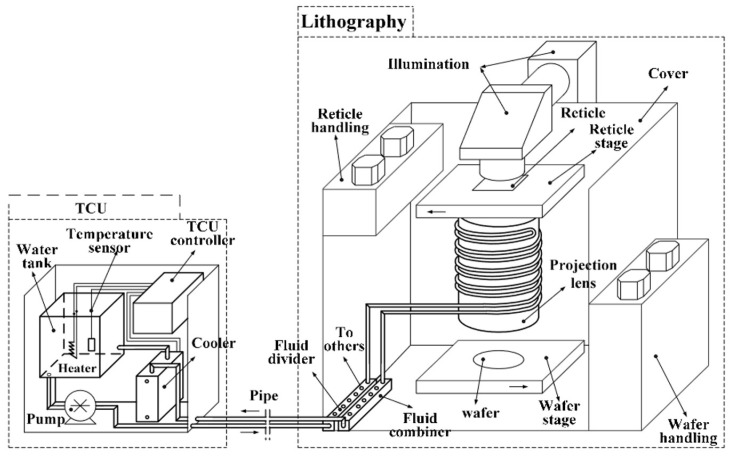
Cooling structure layout for projection lens [49].

Li et al. [49] proposed a modular cooling structure that integrates a temperature control unit (TCU), flow distributors, collectors, and lens water jacket modules. This closed-loop deionized water circulation structure stabilizes thermal load while reducing mechanical vibrations through optimized fluid routing, as shown in Figure 8. The design improves thermal coupling efficiency and structural compatibility, meeting the thermal stability requirements for high-throughput, multi-lens exposure systems.

#### 4.2.2. Infrared Radiation-Based Thermal Control Structures

In EUV lithography, thermal control faces more stringent challenges. Multilayer mirror stacks are used, with typical reflectivity around 70%, while the remaining 30–40% of incident EUV energy is absorbed as heat. Moreover, the system operates under high vacuum, where conventional convective cooling is ineffective. Although some studies proposed coupling the mirror to heat sinks via thermal bridges or liquid-cooled plates to remove average thermal load, practical implementations face several limitations [50]: (1) coolant-induced temperature gradients can introduce parasitic stress and structural deformation, resulting in mirror warping and low-order surface figure errors; (2) liquid flow introduces mechanical disturbances, such as microvibrations, which compromise mirror attitude and positional stability; and (3) water cooling lacks the spatial precision required for localized thermal regulation, often leaving thermal imprints and failing to match the complex heat load distribution on the mirror surface.

**Figure 9:**
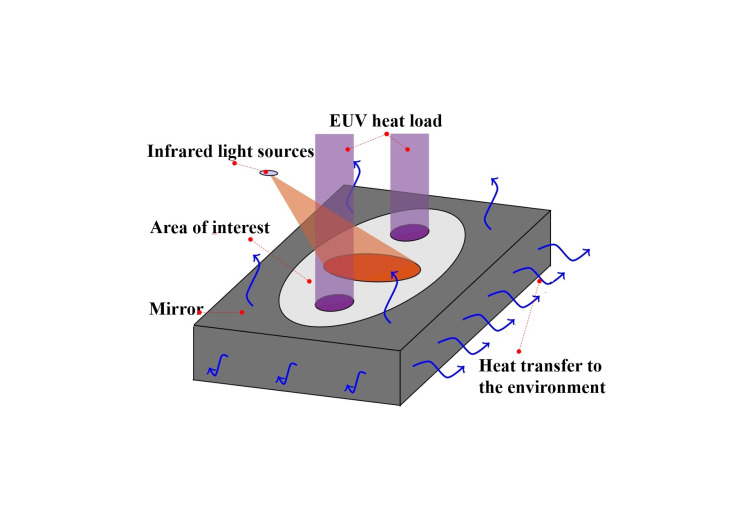
Diagram of infrared-based thermal control structures: IR laser heating of reflective mirror [47].

Building on early developments, EUV lithography systems have adopted similar non-contact IR thermal actuators for localized correction. As illustrated in Figure 9, these actuators are integrated with radiative heat dissipation structures and passive thermal components—such as high-conductivity backplates and thermal connectors—to simultaneously meet the demands of local regulation and overall heat removal. This configuration enables nanometer-scale wavefront control while maintaining global thermal stability across the projection optics [47,50].

Furthermore, related patents propose the use of layered materials with positive and negative thermal expansion coefficients. By applying infrared heating selectively to different material layers, bidirectional compensation of local mirror deformation can be achieved [50].

### 4.3. Thermal Control Algorithms

Control algorithms for projection optics aim to regulate the temperature distribution of mirrors or lenses with high precision, mitigating disturbances caused by exposure power fluctuations and environmental variations. The design of such algorithms depends strongly on the time response characteristics of the thermal control structure and the delay introduced by the feedback path.

#### 4.3.1. Multi-Loop Feedback Control in Water Jacket Systems

Li et al. [49] proposed a hierarchical control strategy based on nonlinear PI regulation for water jacket-cooled DUV projection lens systems. The system is divided into five sequential control stages (D0–D5), with a dual-input dual-output PI controller managing the temperatures of both the lens body and the return coolant. This approach achieves a steady-state thermal regulation precision of ±0.01 °C within 4.5 h.

Building on this, Nie et al. [51] developed a thermal model comprising three coupled subsystems: the proximal thermal medium, the fluid loop, and the lens body. By identifying thermal delays and nonlinear transfer characteristics and calibrating the model with experimental data, the control robustness and predictive accuracy were significantly enhanced.

To further improve dynamic response, Qin et al. [52] introduced a cascaded feedforward–feedback control framework combining MPC with a Smith-predictor-based PID regulator. The MPC module solves a quadratic optimization problem in real time to anticipate future behavior, while the Smith predictor compensates for transport delays in the coolant return path. Additionally, a feedforward path based on laser power disturbance was introduced, reducing temperature overshoot from 0.03 °C to 0.005 °C and shortening the settling time by approximately 40%.

#### 4.3.2. Control Optimization for Infrared Actuation

For thermal compensation of EUV multilayer mirrors, Veldman et al. [47] constructed an infrared excitation control framework based on the patented mirror structure in [50]. Their method involved finite element simulations to obtain temperature rise distributions under 17 representative thermal conditions. Non-negative matrix factorization (NMF) was then applied to extract actuation basis functions, followed by constrained non-convex optimization to determine the minimal heating power required for effective thermal compensation. This strategy enabled mirror thermal regulation within a ±3.5 °C range while reducing the complexity of actuator layout.

### 4.4. Summary and Outlook

This section has reviewed representative thermal regulation structures and control algorithms used in projection optics. In DUV systems, water jacket cooling combined with multi-loop PID controllers and predictive algorithms has achieved steady-state precision at the ±0.01 °C level, well-suited for global temperature control under low-frequency thermal disturbances. In EUV systems, non-contact IR heating paired with radiative dissipation has emerged as a promising solution. The framework developed by Veldman et al., which integrates finite element modeling, NMF-based dimensionality reduction, and nonlinear optimization, successfully compensates for mirror thermal deformation within a ±3.5 °C range while reducing system complexity.

Thermal control in projection optics has evolved from single-mode cooling schemes into multilayered systems optimized through joint structural and algorithmic design. Despite considerable progress in control precision and response time, several issues remain. First, water jacket systems respond sluggishly to rapid or spatially non-uniform thermal disturbances, making them unsuitable for the sub-second control requirements of future high-throughput exposure nodes. Second, while IR-based compensation offers local control capability, it heavily relies on accurate a priori thermal load models and precomputed mirror response functions, lacking online adaptivity and disturbance identification.

Future research should therefore focus on enhancing the adaptivity of infrared compensation systems, developing hybrid thermal load prediction mechanisms, and designing distributed actuator layouts with higher spatial resolution. These directions are key to further advancing the thermal performance and imaging stability of next-generation projection optics.

## 5. Thermal Control of Immersion Liquid Systems

### 5.1. Thermally Induced Errors and Challenges

**Figure 10:**
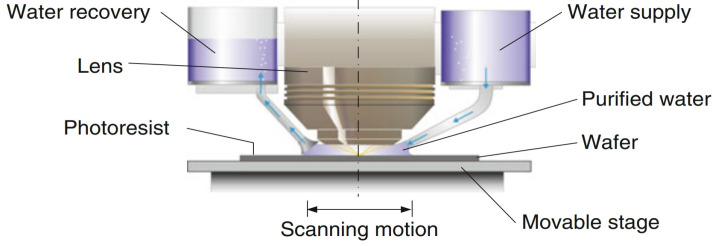
Schematic of the immersion unit position in the lithography system [58].

Immersion lithography primarily employs two modes of liquid supply and recovery: full-wafer immersion and localized liquid fill. Among these, the localized injection method—where liquid is confined to the area between the projection lens and the wafer—has become the mainstream approach due to its lower contamination risk and superior overlay performance [53,54]. As shown in Figure 10, the immersion liquid is confined to the region beneath the projection lens and above the wafer surface, maintained by an immersion hood (IH) that co-moves with the wafer stage. The overall location of the immersion unit within the lithographic system is illustrated in Figure 10.

At the 193 nm wavelength, ultrapure water (UPW) exhibits strong thermorefractive behavior, with a temperature coefficient of refractive index $dn/dT=-1.0\times 10^{-4}\;{}^{\circ}C^{-1}$. Consequently, even mK-level temperature fluctuations can induce focus shifts and wavefront aberrations, significantly affecting critical dimension (CD) control and imaging quality [55,56]. To satisfy the refractive index stability requirement of ±10 ppm, the immersion liquid temperature must be maintained within ±0.1 °C. In more advanced systems such as the 1900i platform, long-term thermal stability of ±0.0025 °C over 1.5 h has already been achieved [57].

In this context, the thermal control system is tasked with regulating the temperature of the liquid along the injection pathway, particularly at the immersion hood outlet. The primary objective is to ensure thermal uniformity and refractive index stability in the lens-to-wafer flow field.

Achieving this level of thermal precision presents significant challenges. First, the immersion liquid temperature is influenced by complex and multi-stage thermal disturbances. These include fluctuations in raw water supply, thermal dynamics introduced during purification and degassing, heat exchange along long-distance piping, and variations arising from multi-channel flow regulation. Together, these factors introduce perturbations through internal heat generation, environmental interaction, and dynamic thermal loads along the liquid distribution path. Second, the thermal control system must suppress all of these disturbances while maintaining temperature stability at the mK level. This requirement is especially demanding because the immersion liquid resides directly within the optical path, where even minor thermal inhomogeneities can induce wavefront aberrations and focus shifts, ultimately degrading imaging fidelity and overlay accuracy.

### 5.2. Thermal Control Structures

To maintain sub-0.01 °C thermal stability in immersion lithography, researchers have proposed various optimization strategies for the UPW thermal conditioning system. These systems are generally built upon fluidic control loops comprising heat exchangers, servo valves, process cooling water (PCW) flow paths, and feedback sensors.

**Figure 11:**
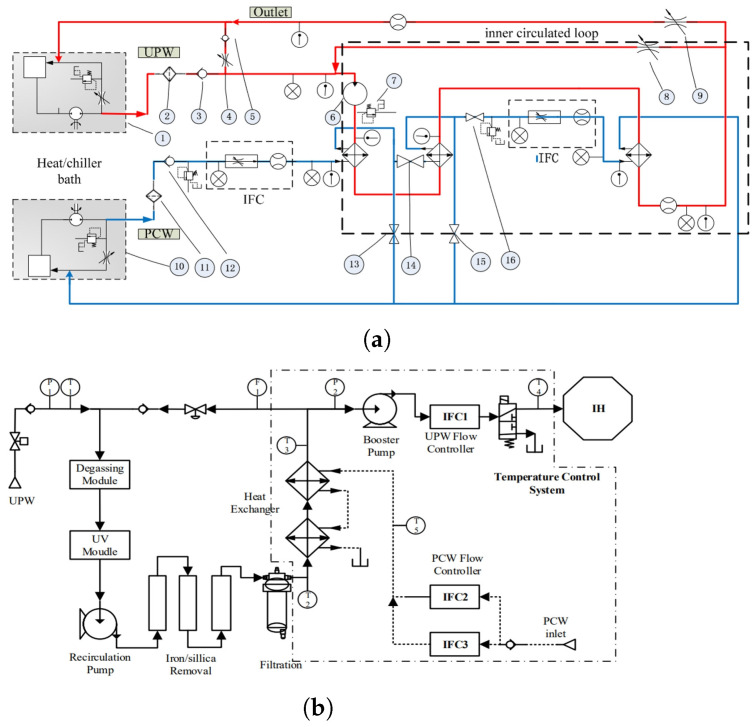
Schematic diagrams of immersion liquid thermal conditioning systems: (**a**) He et al.’s serial dual-exchanger configuration with two-stage servo valve control [59]; (**b**) Li et al.’s parallel regulation architecture using servo valves of different scales [60].

He et al. [59] and Li et al. [60] developed thermal control architectures based on heat exchanger networks regulated by servo valves. As illustrated in Figure 11a, He’s design employs two heat exchangers arranged in series, with dual servo valves providing stepwise regulation of the PCW flow. In contrast, Figure 11b shows Li’s configuration, which adopts a parallel dual-valve setup with differing valve sizes to achieve faster response and finer control of the UPW temperature delivered to the immersion hood. These architectures enable precise liquid temperature control at the injection point, forming a stable and uniform flow field beneath the projection lens during stage scanning.

**Figure 12:**
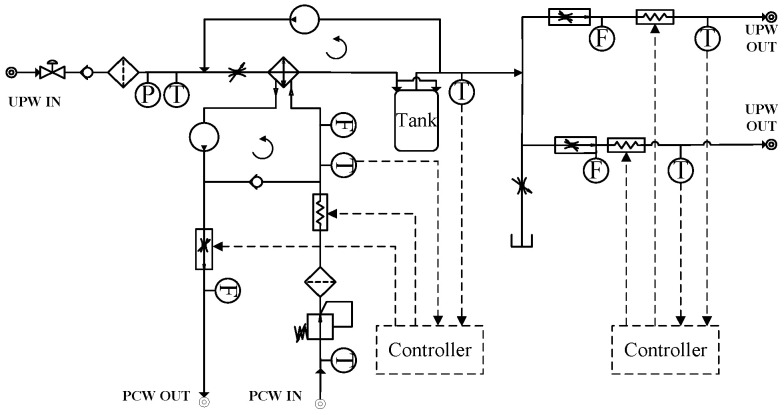
Immersion liquid temperature control structure incorporating circulation loops and a thermal fluctuation attenuation module [61].

To further suppress upstream thermal perturbations, Li and Cao [61] proposed a dual-loop recirculation structure that decouples the UPW and PCW circuits. As shown in Figure 12, a temperature fluctuation attenuation module is positioned directly before the injection bifurcation point. This module reduces the cumulative impact of disturbances originating from source water variation, long-distance piping heat transfer, and flow-channel asymmetry. The resulting thermal damping effect significantly enhances the stability of the immersion liquid temperature at the lens–wafer interface.

### 5.3. Thermal Control Algorithms

The temperature control system for immersion liquid is primarily built upon a constant-temperature circulating water architecture, achieving high-precision regulation through cascade control schemes.

**Figure 13:**
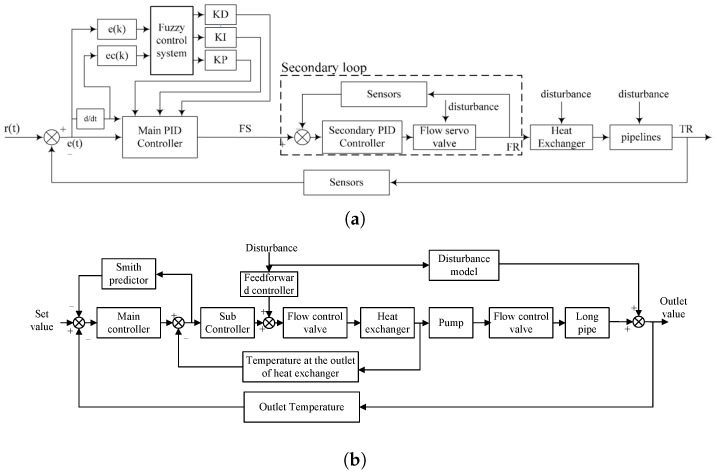
Cascade temperature control systems: (**a**) fuzzy PID-based cascade control [59]; (**b**) enhanced cascade control incorporating feedforward compensation and a Smith predictor [60].

He et al. [59] proposed a fuzzy PID-based cascade control framework, in which the outer loop regulates the UPW temperature setpoint and the inner loop manipulates the PCW flow rate via servo valves. This dual-loop strategy effectively stabilizes the immersion liquid temperature at 22 °C with a deviation of ±0.01 °C, as shown in Figure 13a. The integration of fuzzy logic enhances the system’s responsiveness and precision under small perturbations.

Building upon this foundation, Li et al. [60] introduced an enhanced cascade control architecture that incorporates two key mechanisms: (1) a feedforward compensation module to preemptively counteract environmental disturbances introduced during long-distance pipeline transport; and (2) a predictive model, constructed through system identification, combined with a Smith predictor to compensate for time delays and improve synchronization between the control response and system dynamics. The resulting composite controller integrates separated-integral PI control, feedforward regulation, and lag elements. Experimental results demonstrated superior robustness and dynamic performance, maintaining the system temperature consistently within the ±0.01 °C range (Figure 13b).

**Figure 14:**
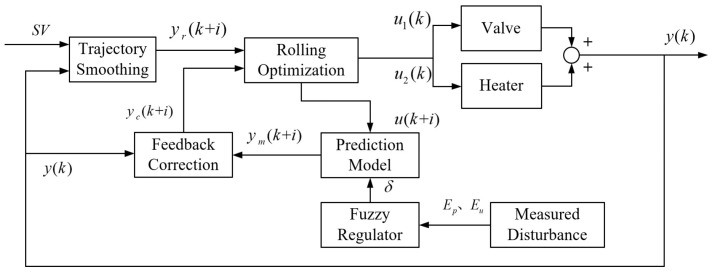
Temperature control architecture of the heating–cooling water circulation system based on the improved DMC strategy, integrating prediction error compensation and a fuzzy rule-based gain adaptation mechanism [62].

To address gain variation and model mismatch caused by dynamic disturbances in UPW and PCW, Li and Cao [62] proposed a temperature control strategy integrating model prediction, error compensation, and a fuzzy rule-based gain adaptation mechanism. As illustrated in Figure 14, the scheme coordinates the actuation of heaters and servo valves through receding horizon optimization. It provides a structured framework for enhancing system adaptability and robustness in immersion temperature control.

### 5.4. Summary and Outlook

Immersion liquid temperature control is a critical enabler for maintaining refractive index stability and imaging precision in advanced lithography systems. In recent years, this domain has undergone significant advancements across both structural design and control algorithms. Structurally, system architectures have evolved from conventional single-loop regulation to more sophisticated configurations involving serial and parallel heat exchangers, dual-loop isolation schemes, and thermal fluctuation attenuation modules. These enhancements effectively suppress multi-source disturbances and improve temperature uniformity at the injection outlet. Algorithmically, control strategies have progressed from basic cascade PID control to integrated schemes that combine feedforward compensation, predictive modeling, and adaptive mechanisms. These developments have markedly improved system robustness and dynamic responsiveness, enabling mK-level temperature stability.

Despite these achievements, several challenges remain. The dynamic and superimposed nature of thermal disturbances is difficult to fully model, and gray-box models based on system identification are prone to accuracy degradation over time. Furthermore, under high-frequency disturbances, current control systems may suffer from limited responsiveness or insufficient adjustment margins. Most existing controllers are still rule-based, offering limited capacity for intelligent optimization and adaptation to evolving operational conditions.

Future research should focus on enhancing online sensing and dynamic modeling through high-resolution temperature and disturbance monitoring, which would support early fault detection and model adaptation. In addition, the integration of machine learning techniques holds promise for real-time parameter tuning and rapid adaptation to nonlinear system behaviors. Finally, a globally coordinated co-optimization framework that unifies hardware design and control algorithm development may enable the next generation of intelligent, performance-driven thermal control systems for immersion lithography.

## 6. Thermal Control of Wafer-Stage Systems

### 6.1. Thermally Induced Errors and Challenges

Thermal deformation of the wafer surface has become a major limiting factor in achieving high-precision imaging in advanced lithography systems. Even minor temperature fluctuations can lead to nanometer-scale geometric distortions, resulting in overlay errors and focus drift. During a typical exposure cycle, the wafer and its supporting structure (wafer table) may undergo temperature variations of up to 1 °C, leading to thermal expansion of approximately 10 nm. Without active compensation, this deformation translates directly into image placement errors [63].

Thermal disturbances arise from several sources: (1) Exposure-induced heating occurs as DUV or EUV illumination deposits energy into the wafer surface, causing local temperature rise and rapid expansion. The magnitude and distribution of this heating are affected by scan trajectory, pattern density, and illumination profile, resulting in highly dynamic, spatially non-uniform heat loads [4,64,65]. (2) Non-exposure-related heat sources, such as Joule heating from stage motors, power dissipation in drive circuits, and ambient radiation, contribute to background thermal drift over time [63]. (3) In immersion lithography, droplet evaporation and redistribution introduce local cooling effects. A significant source is the bubble extraction system (BES), which generates low-pressure flow at the wafer edge to remove gas bubbles. This process causes evaporative cooling of approximately 0.8 °C, leading to edge deformation of up to 11 nm [63,66,67].

The complexity of these errors lies not only in their diverse origins but also in their strong spatial gradients from wafer center to edge and their continuous evolution during scanning. Although current systems such as the XT:1900i achieve temperature uniformity within 0.05 °C across the wafer surface [57], the increasing demands of tighter overlay budgets necessitate further improvement in both spatial uniformity and transient thermal compensation.

Achieving nanometer-level overlay accuracy requires maintaining mK-level temperature stability and uniformity across the wafer throughout the exposure cycle. However, this goal is challenged by several factors: (1) Exposure-induced heating is both non-uniform and dynamically modulated by the scan path and pattern distribution, making it difficult for conventional uniform heating methods to provide timely correction. (2) Evaporative edge cooling in immersion lithography introduces steep thermal gradients that global control strategies cannot fully compensate for, leading to asymmetric deformation that may propagate into the imaging field. (3) Thermal coupling between the wafer and the electrostatic chuck (ESC) must remain stable under vacuum and low-convection conditions. The complex internal architecture of the ESC, including gas channels and embedded electrodes, imposes further constraints on accurate thermal control.

### 6.2. Thermal Control Structures

The thermal control structures of the wafer stage integrate multiple strategies to suppress temperature gradients and dynamically compensate for localized disturbances. Recent research has focused on three major categories of control structures.

#### 6.2.1. Edge-Integrated Sensor-Heater Feedback Structures

**Figure 15:**
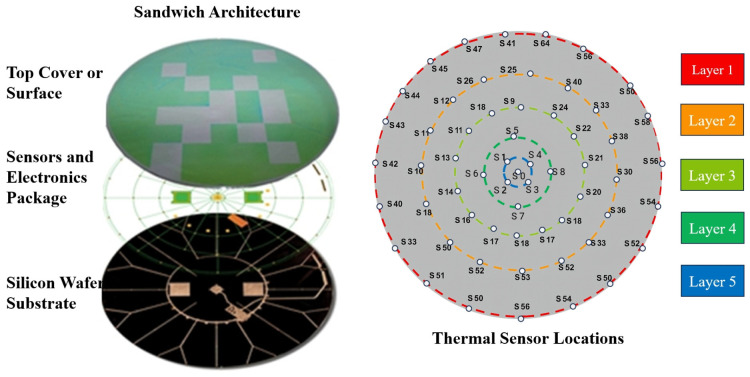
Structure of the wireless SensArray wafer and thermal sensor locations [66].

Edge heating integrated with sensor arrays enables high spatial resolution for local thermal regulation. Ha et al. [66] developed the SensArray wafer system to mitigate thermal non-uniformities caused by immersion liquid evaporation and edge airflow. These non-uniformities can induce overlay errors such as grid scaling and local distortions. The SensArray integrates wireless temperature sensors at both the wafer center and edge, enabling real-time thermal field mapping and precise edge heating compensation. Figure 15 shows the sensor layout.

Considering the evaporation effects of immersion liquid, Heck and Merks et al. [9,63] designed zoned edge heaters for closed-loop compensation of edge cooling effects induced by the BES, significantly improving thermal uniformity. In their approach, distributed temperature sensors are arranged around the periphery of the wafer to monitor local thermal variations in real time, while segmented heaters are placed at the outer edge of the wafer stage to provide localized heating compensation. This design philosophy—positioning sensors and actuators close to the main disturbance sources at the wafer edge—enables precise correction of edge-specific thermal non-uniformities and enhances overall system thermal stability.

#### 6.2.2. Infrared Heating for Scan-Synchronous Compensation

Non-contact IR thermal actuator arrays positioned above the wafer enable scan-synchronous compensation for exposure-induced heating. Veldman et al. [68,69] proposed a radiative heating system, in which spatially modulated IR intensity matches the dynamic heat load of the scanning pattern, allowing fast and localized thermal correction.

#### 6.2.3. Electrostatic Chuck-Based Heat Transfer Structures

The ESC has become the mainstream wafer clamping platform in both DUV and EUV systems due to its excellent planarity, low mechanical stress, and reliable thermal conduction. Electrostatic attraction ensures full-area contact between the wafer and chuck, enhancing thermal coupling. Heat transfer is further assisted by a helium backfill layer introduced between the wafer and chuck surface [70,71,72]. Although structural implementations differ across platforms, all designs aim to maintain a spatially uniform and temporally stable temperature field at the wafer–chuck interface [73,74,75,76,77].

**Figure 16:**
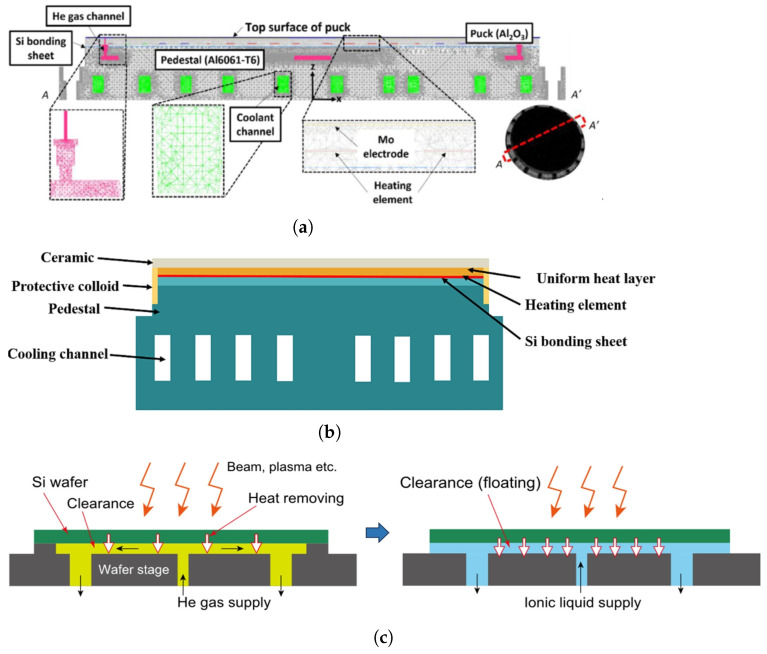
Schematic diagrams of ESC thermal designs, illustrating key innovations: (**a**) multi-zone heating power distribution and the limited impact of increased cooling gas flow on uniformity [79]; (**b**) influence of cooling liquid flow and channel geometry on the temperature field [80]; (**c**) ionic liquid cooling as an alternative to helium gas, improving vacuum compatibility and temperature stability [81].

Ongoing research has addressed further optimization through multi-zone heating, electrode layout refinement, gas flow control, and cooling-channel configuration. Sun et al. [78] examined how electrode arrangement, dielectric layer properties, and gas type influence both thermal uniformity and clamping performance. As shown in Figure 16a, Jang [79] demonstrated that although increasing helium backpressure offers limited benefit, combining it with zoned heating allows for fine-scale thermal tuning, achieving mK-level resolution. As shown in Figure 16b, Liu [80] evaluated the impact of the coolant flow rate, channel pitch, and orientation on the wafer temperature field, showing that geometric optimization of cooling channels reduces gradients and improves uniformity. Finally, as shown in Figure 16c, Okabe [81] proposed ionic liquids as a replacement for helium to enhance thermal conductivity and reduce outgassing, improving performance under vacuum and long-duration operation.

These sensor–actuator–clamping co-optimizations, combining contact-based and radiative methods, represent a multi-dimensional pathway to achieving the mK-level control precision required for future wafer stages in high-NA EUV lithography.

### 6.3. Thermal Control Algorithms

To achieve mK-level temperature control precision across a 300 mm wafer, advanced control algorithms must be introduced to handle dynamic thermal loads, actuator underactuation, and non-uniform heat source distributions. Recent studies have mainly focused on the three model-based control strategies described below.

#### 6.3.1. Zonal Controller for Centre–Edge Thermal Regulation

**Figure 17:**
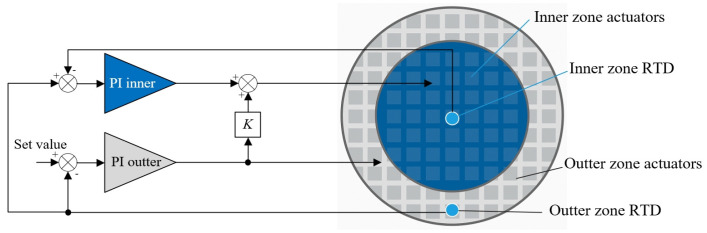
Schematic representation of a zonal wafer temperature control scheme. The left part illustrates the model-based control architecture for achieving across-wafer thermal uniformity, while the right part shows the arrangement of actuators and RTD sensors on the wafer, defining the inner and outer zones [82].

Tay et al. [82] proposed a dual-zone thermal control strategy by partitioning the wafer into central and edge regions, with each regulated by separate controllers coordinating a bottom-side thermal actuator array. The system employs an integrated bake/chill platform with embedded resistance temperature detectors (RTD) positioned at the center zone and edge zone to provide real-time temperature feedback. A hierarchical control logic is adopted: the edge controller performs fast trajectory tracking, while the center controller references edge conditions and applies static feedforward compensation to ensure steady-state uniformity. This architecture enables the system to maintain wafer temperature differentials within ±0.1 °C during thermal cycling. Although the wafer bottom structure in track systems differs from that in exposure stages, the zonal temperature control strategy is still instructive for achieving local thermal uniformity. Based on the design concepts, a modified schematic is redrawn as shown in Figure 17.

#### 6.3.2. Integrated Actuator Design and Input Optimization

Veldman et al. [69] proposed a two-stage optimization framework for the layout and control of infrared thermal actuator arrays above the wafer. In the first stage, the actuator geometry and spatial arrangement are optimized by minimizing wavefront distortion under thermal and optical constraints. The second stage refines the power input profile by penalizing excessive thermal load and introducing regularization and imaging performance constraints to enhance robustness. This integrated approach couples structural design with input optimization, enabling effective compensation of exposure-induced heating and improving sub-nanometer thermal control performance.

#### 6.3.3. Deformation-Based State Estimation and Control

Heck et al. [9] developed a thermo–structural–optical control strategy that directly suppresses wafer deformation rather than merely regulating temperature. A high-dimensional finite element model is reduced using frequency response function (FRF) techniques to construct a low-order dynamic observer. An extended Kalman filter is then applied to estimate the deformation states, which are fed into a linear quadratic regulator (LQR) to actively compensate for distortion through closed-loop heater control. This controller achieves significant improvements, even under underactuated conditions. Experimental results show that, compared with conventional thermal controllers (maintaining <5mK variation), the deformation-based controller reduces edge deformation by approximately 60% without requiring additional sensors or actuators.

### 6.4. Summary and Outlook

Wafer-stage thermal control serves as a critical enabler for achieving sub-nanometer imaging accuracy in advanced lithography systems. The primary challenges stem from the spatial non-uniformity and temporal variability of heat sources. As technology nodes advance, conventional global isothermal strategies have evolved into a multilayered regulation framework that integrates local disturbance sensing, active compensation, and model-driven control.

In terms of structural design, these systems have progressed from global passive cooling to collaborative schemes, including center–edge zonal regulation, exposure-synchronized compensation, and enhanced thermal coupling at the wafer interface. Notably, multi-zone control at the ESC interface has pushed local temperature uniformity below the 10 mK level, laying a robust foundation for stable thermal control.

On the algorithmic front, current mainstream approaches focus on zonal temperature control architecture, accurate and reduced-order thermomechanical modeling, and optimized control allocation strategies.

Despite the capability of existing solutions to suppress thermal deformation under mK-level temperature gradients, several limitations remain: the system still suffers from response latency to local disturbances and limited accuracy in high-dimensional model identification.

Future development of wafer-stage thermal control will rely on the coordinated optimization of thermal field sensing, actuation precision, and predictive control to ensure the thermal stability and image consistency required by next-generation lithography. Lastly, unlike previous studies that primarily target a single dominant disturbance, real exposure scenarios present a combination of interacting heat sources. Therefore, developing full-field thermal disturbance models and validating control strategies under realistic operating conditions is essential, as current research often lacks comprehensive multi-variable case studies.

## 7. Thermal Control of Metrology Systems

### 7.1. Thermally Induced Errors and Challenges

In advanced lithography systems, the metrology subsystem comprises laser interferometers, grating interferometers, and encoder arrays, enabling high-precision, multi-degree-of-freedom displacement measurements and closed-loop position control [83]. Among these, interferometers play a pivotal role in determining stage position and overlay accuracy. Since the optical path length and structural geometry are both sensitive to temperature variation, thermal control has become essential for maintaining sub-nanometer measurement precision.

**Figure 18:**
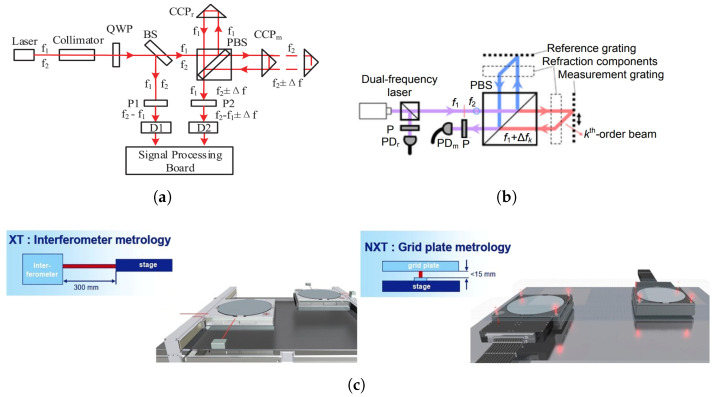
Representative interferometric metrology systems used in lithographic wafer-stage control: (**a**) schematic of a laser interferometer [84]; (**b**) schematic of a grating interferometer [85]; (**c**) schematics of a conventional interferometer system with long, variable beams and a new encoder system with short, fixed-beam interferometers [86].

Laser interferometers, as shown in Figure 18a, rely on a dual-frequency laser beam split into reference and measurement paths [84]. The measurement beam reflects off a retroreflector mounted on the moving stage, and upon recombination with the reference beam, produces interference fringes from which displacement is derived. However, as predicted by the Edlén equation, fluctuations in air temperature affect the refractive index, while thermal expansion of optical mounts introduces additional path-length errors, both potentially inducing nanometer-scale measurement drift. To mitigate these effects, interferometers must operate within a thermally regulated environment, typically controlled to within mK-level stability. Moreover, long travel paths on the wafer stage are particularly vulnerable to ambient air gradients, making spatial thermal uniformity along the beam path a critical challenge.

As illustrated in Figure 18b, grating interferometers offer an alternative approach by using a compact optical path and diffraction-based measurement, as reviewed by Hu et al. [85]. They typically comprise a dual-frequency laser source, a fixed reference grating, a moving measurement grating mounted on the wafer stage, and an integrated readhead detector. The interference signal generated by overlapping diffracted and reference beams is processed via heterodyne detection to yield six-degree-of-freedom motion information. While the short beam path reduces sensitivity to ambient disturbances, the readhead is mounted directly on the moving stage and undergoes rapid scanning, exposing it to heat sources such as the wafer stage and surrounding airflow. Therefore, thermal control must ensure uniformity and stability in the optical path region during high-speed motion, where temperature gradients may intrude dynamically.

In summary, thermal control of interferometers faces dual challenges: on the one hand, the entire measurement system must be maintained in a stable, temperature-controlled environment to reduce the influence of ambient temperature fluctuations on the optical path; on the other hand, high spatial uniformity and mK-level stability of the air temperature along the optical path must be achieved to suppress measurement errors caused by optical path-length variations. For the two types of interferometers already employed in advanced lithography tools, as shown in Figure 18c [86], the thermal control difficulties also differ. Laser interferometers feature long optical paths, with the main challenge being how to maintain consistent temperature fields along the entire path. In contrast, although grating interferometers have shorter optical paths, the readhead moves rapidly with the wafer stage and is susceptible to external thermal disturbances, posing more complex challenges in local dynamic thermal non-uniformity. Addressing these issues, achieving stable and reliable thermal control has become one of the key bottlenecks in high-precision interferometric metrology systems.

### 7.2. Thermal Control Strategies for Interferometers

To address the issue of long-path laser interferometers being highly susceptible to ambient thermal fluctuations, various local air delivery techniques have been proposed to stabilize the temperature field in both the wafer surface and interferometric beam paths. For instance, Vogel et al. [87] from ASML introduced a horizontal laminar airflow approach targeting the wafer and beam-path regions of the lithography tool. By employing non-uniform perforated plates to distribute flow, this method ensures a uniform airflow field at the outlet and suppresses turbulence within the gas bath. As a result, thermal exchange between the gas environment and ambient air is minimized, maintaining uniform temperatures over the wafer and optical paths. Van der Ham et al. [88] further proposed a dual-layer gas plenum design for the lithography tool. The upper chamber uses flow regulators to condition the uniformity and pressure of the incoming temperature-controlled gas, while the lower chamber ensures evenly distributed delivery to multiple outlets, enhancing the uniformity of the local gas temperature field. Liu et al. [89] proposed a directional airflow system that dynamically adjusts the orientation of the local gas stream to follow the motion of the wafer stage, thereby maintaining thermal uniformity over the interferometric beam path and wafer region in real time.

**Figure 19:**
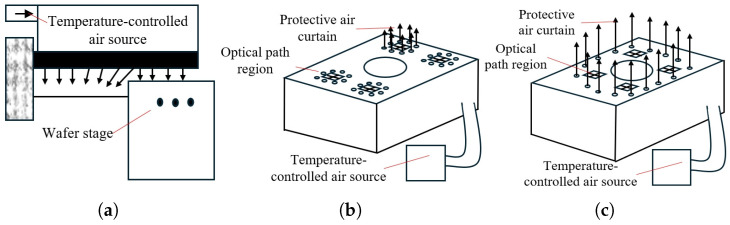
Representative local gas delivery structures for interferometric thermal control: (**a**) conventional laminar-flow gas curtain over wafer and beam paths [87]; (**b**) individual circular gas curtains for separate beam-path protection [90]; (**c**) shared circular gas curtain enclosing all beam paths [90].

For grating interferometers, the readhead and its associated beam path are key targets of thermal regulation. Conventional local laminar airflow systems (see Figure 19a) are often employed to stabilize the ambient temperature around the stage and optical paths. However, due to the open-flow nature of these systems, they are prone to turbulent interference from high-speed stage motion, which compromises their thermal shielding performance. To address this limitation, Luttikhuis et al. [90] proposed a local shielding method using circular gas curtains. Two configurations were demonstrated: one using individual gas curtains for each beam path (see Figure 19b), and another employing a shared gas curtain to enclose all optical paths (see Figure 19c). This shielding concept shows strong potential for thermally isolating the readhead optical paths from environmental airflow disturbances while preserving compact system integration.

### 7.3. Summary and Outlook

Currently, local air-shower systems have proven effective in improving the temperature uniformity along the extended optical paths of laser interferometers. However, due to the open-space nature of these systems, the airflow is easily disrupted by the high-speed motion of the wafer stage, making the temperature field vulnerable to external thermal disturbances. Further refinement of laminar airflow design is therefore essential to suppress turbulence in the local flow field and enhance thermal uniformity around the optical path.

For grating interferometers, while localized water cooling of the readhead and circular air curtains around the optical paths provide feasible active thermal control solutions, single-layer gas curtains exhibit limited shielding capability against high-speed perturbations. Moreover, the high-velocity gas flow may induce vibration in the reference grid plate. To address these limitations, future efforts should focus on the design of multilayer gas curtain structures to improve isolation from external airflow, enhance thermal uniformity, and simultaneously reduce gas velocity to mitigate excitation of grid plate vibrations.

In addition to optimizing thermal field uniformity, both the local air-shower systems in laser interferometers and the gas curtain shields in grating interferometers fundamentally rely on precise thermal sources capable of delivering ultra-stable airflow. Therefore, the development of air supply systems capable of regulating airflow temperature at the mK-level will be a critical research direction going forward.

## 8. Conclusions and Outlook

This paper presents a comprehensive review of thermal control strategies in advanced DUV and EUV lithographic systems, encompassing critical subsystems including the light source, projection optics, immersion module, wafer stage, reticle system, and metrology system. Focusing on thermally induced errors, structural temperature regulation, and control algorithm development, we systematically summarize the current state of research and representative implementations, outlining the engineering logic behind achieving sub-nanometer overlay and CD control.

Substantial progress has been achieved in both the structural and algorithmic dimensions of thermal control. On the structural side, dual-loop liquid temperature regulation, infrared compensation, and integration of thermomechanical actuators have significantly improved temperature uniformity and distortion suppression. On the control side, the system has evolved from traditional PID and fuzzy PID cascade architectures to advanced hybrid frameworks incorporating MPC, adaptive feedforward–feedback coordination, and gain self-tuning strategies. These developments have enabled milli-Kelvin-level thermal precision with enhanced robustness.

Notably, the wafer stage and reticle system exhibit strong similarity in thermal error sources, structural configurations, and control objectives. Both are subject to intense localized heating during exposure and require mK-level temperature stability and spatial thermal uniformity. They also share thermal contact structures such as ESC, center–edge zonal heating, and layouts that integrate local actuators with distributed sensing. However, most existing research remains confined within individual subsystems, limiting the potential for cross-module thermal modeling, sensor deployment, and coordinated control architectures.

Furthermore, in addition to thermal control structures and algorithms, thermal sensing technologies are critical yet often overlooked elements that directly impact feedback quality and control-loop performance. High-resolution sensors (e.g., SPRTs with resistance bridges) provide mK-level accuracy, while dense spatial arrays enable localized thermal field monitoring essential for fine-grained correction. Improved sensing integration and faster response enhance control bandwidth and disturbance rejection, and future lithography systems will increasingly rely on these advancements to achieve robust sub-milli-Kelvin stability and overlay precision.

Looking ahead, further advances in thermal control for lithographic tools are anticipated in the following directions:

(1) Unified cross-module thermal control architectures: Future efforts should move beyond traditional subsystem boundaries to establish system-level thermal modeling frameworks and control schemes. This would enable sensor information sharing and actuator coordination across modules, improving overall thermal stability and reducing inter-field non-uniformity-induced imaging errors.

(2) Hybrid modeling combining physics and data-driven approaches: By leveraging high-density temperature sensor arrays and process-side feedback, gray-box models or digital twins can be established for real-time prediction and compensation of local thermal drifts while enhancing adaptation to nonlinear dynamics.

(3) Intelligent and adaptive control mechanisms: Control strategies that combine MPC with gain scheduling, adaptive feedforward compensation, and spatial feedback loops will offer stronger robustness and generalizability. Reinforcement learning in simulation environments may provide promising solutions for self-optimized control under complex thermal fields.

(4) Advanced thermal structure design through co-optimization: High-conductivity and low-CTE materials, topology-optimized cooling channels, and additive manufacturing can be used to realize compact and responsive local thermal structures. Integrating embedded sensors with multi-point heaters will further enhance responsiveness and compensation capacity.

Through the co-optimized development of structures and algorithms, lithographic systems are expected to achieve the ultra-high thermal stability required for 2 nm and beyond technology nodes. Enhanced thermal control capabilities will become a foundational enabler for the continuation of Moore’s Law and the advancement of next-generation semiconductor manufacturing.

## Data Availability

No new data were created or analysed in this study. Data sharing is therefore not applicable to this article.
